# Antibiofilm and Protein-Repellent Polymethylmethacrylate Denture Base Acrylic Resin for Treatment of Denture Stomatitis

**DOI:** 10.3390/ma14051067

**Published:** 2021-02-25

**Authors:** Salwa O. Bajunaid, Bashayer H. Baras, Abdulrahman A. Balhaddad, Michael D. Weir, Hockin H. K. Xu

**Affiliations:** 1 Department of Prosthetic Sciences, College of Dentistry, King Saud University, Riyadh 60169-15, Saudi Arabia; bajunaid@ksu.edu.sa; 2 Department of Restorative Dental Sciences, College of Dentistry, King Saud University, Riyadh 60169-15, Saudi Arabia; 3 Department of Advanced Oral Sciences and Therapeutics, University of Maryland School of Dentistry, Baltimore, MD 21201, USA; aabalhaddad@umaryland.edu (A.A.B.); michael.weir@umaryland.edu (M.D.W.); hxu2@umaryland.edu (H.H.K.X.)

**Keywords:** fungal retarding, biofilm adhesion, *Candida albicans*, denture stomatitis, protein repellent, solution pH, surface roughness

## Abstract

*Candida albicans* (*C. albicans*) biofilm is a common etiological factor in denture stomatitis. The purpose of this study was to investigate the effects of incorporating 2-methacryloyloxyethyl phosphorylcholine (MPC) as a protein repellent into a new high-impact denture acrylic (HIPA) resin on the surface roughness, solution pH, and *C. albicans* biofilm adhesion to the denture base. The new acrylic denture resin base was formulated by mixing MPC into HIPA resin at mass fractions of 1.5%, 3%, and 4.5%. Surface roughness was measured using a Mitutoyo surface roughness tester. *C. albicans* biofilm growth and viability were assessed via colony forming unit counts. The pH of the biofilm growth medium was measured using a digital pH meter. Adding MPC to the HIPA resin at percentages of 1.5% and 3% increased the roughness values significantly (*p* < 0.05), while adding 4.5% MPC resulted in no difference in roughness values to that of the control group (*p* > 0.05). All experimental groups demonstrated neutral pH values (pH ≅ 7) and were not significantly different from each other (*p* > 0.05). Incorporating 2-methacryloyloxyethyl phosphorylcholine at 4.5% resulted in a significant (≅1 log) colony-forming unit reduction compared with the control group with 0% MPC (*p* < 0.05). A fungal-retarding denture acrylic resin was developed through the incorporation of MPC for its protein-repelling properties. This newly developed denture acrylic material has the potential to prevent oral microbial infections, such as denture stomatitis.

## 1. Introduction

In dentistry, partial or complete edentulism among the elderly remains a significant problem, as it affects nutrition, mastication, pronunciation, and overall quality of life (QoL). A common treatment modality for the replacement of missing teeth of these patients is the use of removable dental prosthesis [1]. However, a prevalent pathologic condition among denture wearers is denture stomatitis (DS), which was shown to affect up to 72% of complete denture wearers [2]. Denture stomatitis is a multifactorial inflammatory condition with an unknown specific etiology. Although the literature shows that the most common cause of DS is microbial biofilms, other factors that contribute to developing this condition include ill-fitted dentures, long-duration denture use, poor oral hygiene, xerostomia, and some systemic conditions such as diabetes [3,4,5].

Microorganisms found on the surface of dentures are organized in a complex biofilm structure composed mainly of an extracellular polymeric substance (EPS). The EPS is composed of microbial components, salivary proteins, polysaccharides, nucleic acids, and other biopolymers [6]. Microorganisms enclosed in EPS have shown to be 1000-fold more resistant to host defense cells and antimicrobial agents (for example, disinfectants and antibiotics) than their planktonic counterpart [7].

*Candida albicans* (*C. albicans*) biofilms have been commonly associated with the development of denture stomatitis. The colonization of *C. albicans* is mainly attributed to the rough surface texture of dentures and the reduced salivary flow beneath the denture surface, which favors *C. albicans* biofilm formation and proliferation [8,9]. In a previous study conducted by Ramage et al., scanning electron microscopy analysis of samples of denture acrylic retrieved from patients with denture stomatitis revealed a dense Candida biofilm especially along cracks and rough denture surfaces [4].

Denture stomatitis associated with Candida infections is commonly seen on the palatal mucosa in areas in contact with the denture surface. Mandibular mucosa may also be affected, however, less frequently. It is clinically represented by pinpoint hyperemia, diffuse erythematous, or granular/papillary type [8]. Although lesions may be asymptomatic, patients may experience soreness and a burning sensation, which may negatively impact their mastication, subsequently resulting in limited nutritional intake [8].

Due to its high prevalence, several treatment strategies have been proposed to treat DS. Some of these treatments include the use of local and systemic fungal-retarding drugs such as nystatin and miconazole [10,11,12], and denture disinfectants such as chlorhexidine gluconate, sodium hypochlorite, and hydrogen peroxide [13,14]. Other efforts have been made to produce surface modifications of the denture acrylic resin to reduce the adhesion of *C. albicans* and biofilm formation. These materials have been mainly used to increase the surface hydrophilicity of the denture acrylic resin and reduce the surface energy, all of which result in reduced *C. albicans* adhesion and colonization on the denture surface. Some of these materials include 2-octyl cyanoacrylate [15], silane-silicon dioxide (SiO_2_) [16], and photo-polymerized coatings containing zwitterion or hydrophilic monomers [17], all of which produced favorable effects in inhibiting *C. albicans* biofilms.

2-methacryloyloxyethyl phosphorylcholine (MPC) is a methacrylate with a phospholipid polar group in its side chain [18]. Due to its biocompatibility and hydrophilicity, MPC has previously demonstrated potent protein-repelling and antiadhesion properties [19]. In previous studies, when MPC was incorporated into dental composites and adhesives, it demonstrated a considerable reduction in protein adsorption and bacterial attachment without compromising the other needed mechanical, physical, or bonding properties [20,21].

To date, there has been no report on the incorporation of MPC into a new high-impact pour polymethylmethacrylate self-cure denture base acrylic resin (Lucitone HIPA, Dentsply Sirona) and the potential benefits of its use in treating DS. The purpose of this study was to investigate the effects of incorporating different concentrations of MPC into high-impact denture acrylic (HIPA) resin on the surface roughness, solution pH, and *C. albicans* biofilm adhesion. Three hypotheses were tested: (1) incorporating MPC into the HIPA acrylic resin would not jeopardize the surface roughness properties; (2) addition of MPC would not change the pH and the acidity of the resin and hence the adherence of *C. albicans* to the resin; (3) MPC-containing acrylic resin would greatly reduce colony-forming unit (CFU) counts compared to the control group.

## 2. Materials and Methods

Lucitone HIPA, a high-impact pourable denture acrylic resin (Dentsply Sirona, York, PA, USA) was used as the carrier for the protein-repellent monomer, 2-methacryloyloxyethyl phosphorylcholine (MPC). It is a self-cure denture base acrylic (polymethylmethacrylate) used for fabrication, repair, relining, or rebasing of full or partial dentures. 

MPC was purchased from Sigma-Aldrich (Sigma-Aldrich, St. Louis, MO, USA).  MPC was mixed into the HIPA resin at mass fractions of 1.5%, 3%, and 4.5%. The purpose was to investigate the lowest concentration of MPC that could be added to the HIPA resin to produce the highest protein-repelling properties without jeopardizing the surface roughness properties. HIPA resin with 0% MPC was used as the control group for comparison. The following groups were tested for their surface roughness, effect on solution pH, and fungal-retarding properties:

(1) HIPA resin + 0% MPC (control);
(2) HIPA resin + 1.5% MPC;
(3) HIPA resin + 3% MPC;
(4) HIPA resin + 4.5% MPC.

Acrylic resin disks were fabricated by pouring the tested material into 5 mm × 2 mm circular molds placed between two metal plates to create a smooth surface and avoid any variations in the surface texture. All disks were placed in a pressure pot at 20 psi (1.4 bars) at a temperature adjusted to 113 ± 2 °F to be cured. Surface roughness measurements were obtained from a Mitutoyo surface roughness tester (SJ-310, SURFTEST, Kanagawa, Japan). Surface roughness (Ra) averages were obtained and calculated. Disks with a 5 mm × 2 mm dimension were fabricated and heat-cured in a pressure pot at 20 psi (1.4 bars). After curing, all disks were sterilized with ethylene oxide (AnproleneAN 74i, Andersen, Haw River, NC, USA) and de-gassed for 3 days. The use of *C. albicans* (SC5314) (American Type Culture) was approved by the University of Maryland. 

Brain heart infusion (BHI) broth (Sigma-Aldrich) supplemented with 0.5% glucose was used as the growth medium. First, 25 µL from the *C. albicans* stock culture was added into 10 mL of the glucose-supplemented BHI media and was incubated for 24 h at 37 °C with 5% CO_2_. After 24 h, 1 mL from the *C. albicans* inoculum was added into 19 mL glucose-supplemented BHI and vortexed. Acrylic resin disks were placed in a 24-well plate, and 1.5 mL of the inoculum was added to each well. Samples were then incubated at 5% CO_2_ and 37 °C for 24 h. After that, disks were transferred into a new 24-well plate filled with 1.5 mL of fresh BHI media and incubated for an additional 24 h. This allowed the formation of a relatively mature *C. albicans* biofilm on the acrylic resin disks. 

After formation of the *C. albicans* biofilms on the acrylic resin as described above, the biofilms on the disks were harvested in phosphate-buffered solution (PBS) by sonication and vertexing (Fisher, Pittsburg, PA, USA). The harvested biofilm suspension was then serially diluted and dispersed onto BHI agar plates. All plates were incubated for 24 h at 37 °C in 5% CO_2_, and the number of colonies were counted using a CFU plate reader (Reichert, Depew, NY, USA). The number of colonies was used along with their dilution factor to determine the CFU counts.

The 2-day biofilm growth medium was measured for its pH to assess whether MPC could cause any changes in medium acidity. The pH of the biofilm growth medium was measured using a digital pH meter (Accumet XL25, Thermo Fisher Scientific, Waltham, PA, USA). Before each pH measurement, the pH meter was calibrated at pH 4, 7, and 10 using commercial standard solutions.

Statistical analysis was performed with SPSS 20 [22] (SPSS) at α = 0.05. A one-way analysis of variance (ANOVA) test was performed to determine the mean differences between the groups. Tukey’s multiple comparison test was also utilized.

## 3. Results

**Figure 1:**
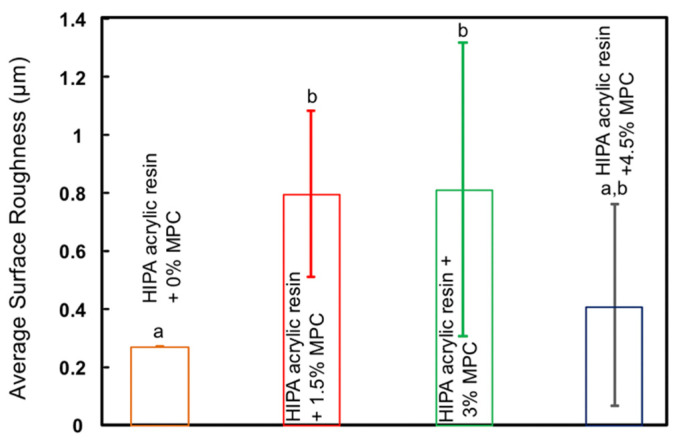
Average roughness values of high impact denture acrylic (HIPA) resin mixed with 0%, 1.5%, 3%, and 4.5% % 2-methacryloyloxyethyl phosphorylcholine (MPC) as a protein repellent (mean ± SD; n = 12). HIPA resin with 4.5% MPC showed surface roughness values similar to that of the control group (*p* > 0.05).

The average roughness values of all groups are plotted in Figure 1 (mean ± SD; n = 12). When MPC was added to the HIPA at 1.5% and 3%, the roughness values significantly increased (*p* < 0.05) compared to the control group with 0% MPC. However, increasing the MPC percentage to 4.5% resulted in roughness values that were not different than that of the control group (*p >* 0.05). 

**Figure 2:**
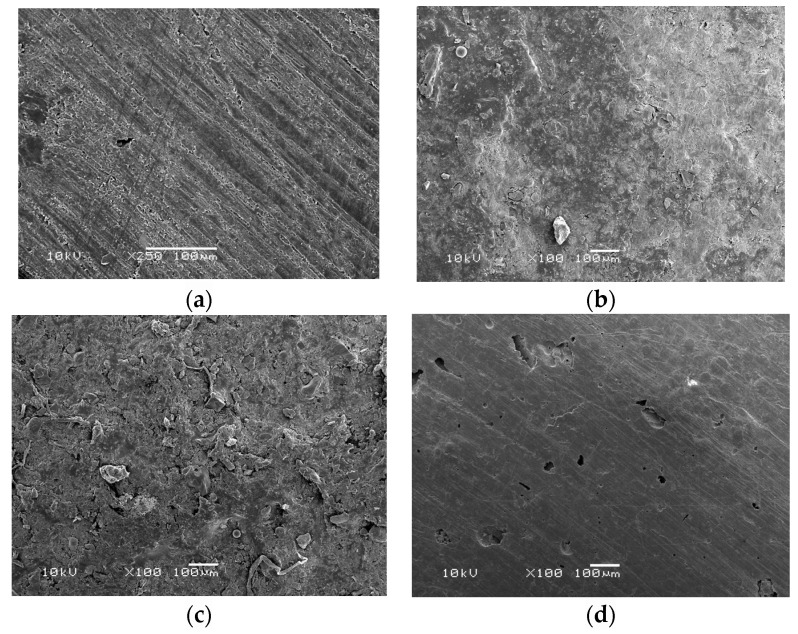
Scanning electron microscopy (SEM) showing surface roughness of acrylic samples mixed with: 0% MPC (**a**), 1.5% MPC (**b**), 3% MPC (**c**), and 4.5% MPC (**d**) as a protein repellent at 100× magnification. HIPA acrylic resin with 4.5% MPC showed a surface roughness image comparable to that of the control group.

These results were also supported by the scanning electron microscopy (SEM) images (Figure 2), where the incorporation of 4.5% MPC showed a smoother surface that was comparable to that of the control group.

**Figure 3:**
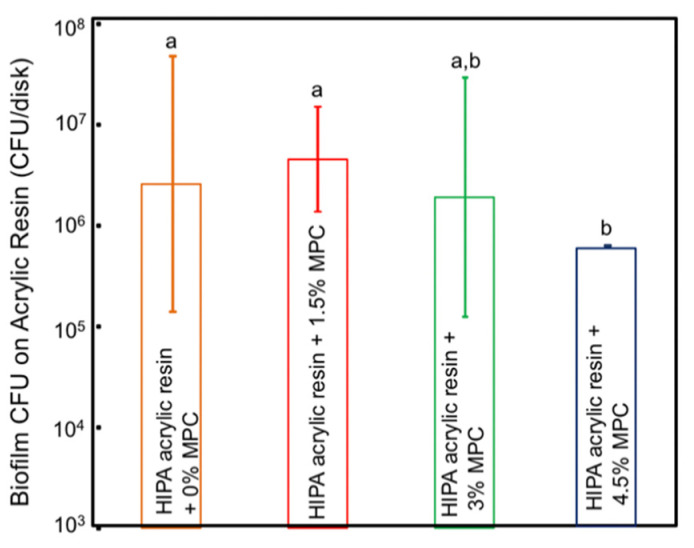
Colony-forming unit (CFU) counts of the *C. albicans* 2-day biofilm on the HIPA acrylic disks (mean ± SD; n = 4). The experimental group with 4.5% MPC resulted in a significant (≅1 log) CFU reduction compared to the control group with 0% MPC (*p* < 0.05).

Figure 3 presents the CFU counts of the *C. albicans* 2-day biofilm on the HIPA disks (mean ± SD; n = 4). Incorporating MPC at 1.5% and 3% did not significantly reduce the CFU counts compared to HIPA control group with 0% MPC (*p >* 0.05). However, increasing the MPC percentage to 4.5% resulted in a significant (≅1 log) CFU reduction compared to the control group with 0% MPC (*p* < 0.05). 

**Figure 4:**
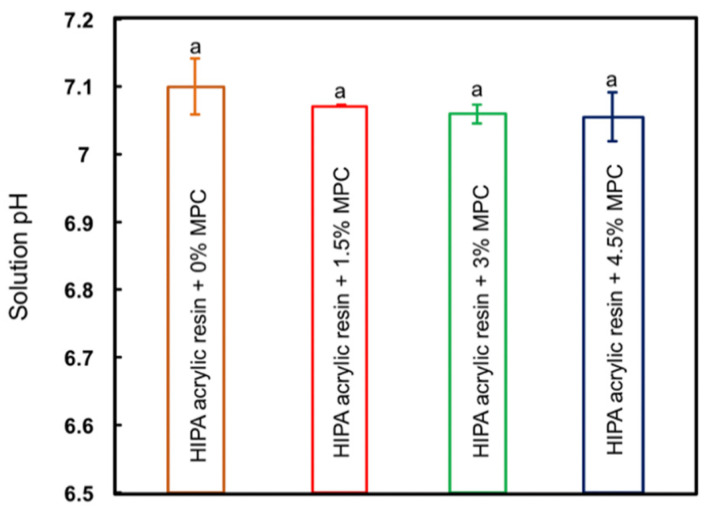
Solution pH values of HIPA groups with MPC at 0%, 1.5%, 3%, and 4.5% (mean ± SD; n = 4). All groups demonstrated solution pH values that were not significantly different from each other (pH ≅ 7) (*p >* 0.05).

The effects of incorporating MPC into the HIPA on solution pH are plotted in Figure 4 (mean ± SD; n = 4). Compared to the HIPA acrylic control with 0% MPC, all experimental groups with MPC percentages ranging from 1.5% to 4.5% presented solution pH values that were not significantly different from each other (*p >* 0.05). All groups demonstrated neutral pH values (pH ≅ 7). Incorporating MPC into HIPA produced no significant effect on solution pH. 

## 4. Discussion

In the present study, we developed an antibiofilm acrylic denture resin through the incorporation of MPC, known for its protein-repelling properties. Incorporating MPC at a weight percentage of 4.5% into a commercially available Lucitone high-impact denture acrylic resin reduced biofilm CFU by one order of magnitude without jeopardizing the roughness properties. In addition, the incorporation of MPC did not alter the acidity of the surrounding environment, which could favor biofilm formation and proliferation.

Denture stomatitis is a common oral condition among individuals wearing a removable dental prosthesis [1,23,24]. DS has previously been shown to affect one in every three removable-denture wearers [23]. Although the cause of DS has been identified as multifactorial, fungal microorganisms, particularly *C. albicans*, have demonstrated a special relevance to DS [4,25]. The ability of *C. albicans* to adhere to the fitting surface of acrylic dentures and to induce biofilm formation has been well-established, especially in patients with poor oral hygiene [3,23,25].

According to the literature, oral proteins adsorbed into oral surfaces favor the adhesion and colonization of oral microorganisms. These proteins preferentially adsorb to hydrophobic surfaces. Previous studies showed the adhesive ability of Streptococcus mutans (*S. mutans*) on denture acrylic resin [26]. The adherent *S. mutans* on the denture acrylic promotes the colonization of candida cells through the production of extracellular polymeric substance [26,27]. In addition, the production of lactic acid by *S. mutans* as a metabolic by-product further favors the growth of fungal species [27].

Developing antimicrobial and protein-repelling agents has gained the attention of scientists in a wide range of specialties. Surface alterations to produce hydrophilic surfaces have been shown to decrease protein adsorption and subsequent microbial attachment. For example, medical devices with protein-repelling properties have been used in artificial blood vessels, implantable artificial hearts, and artificial lungs [28]. In dentistry, protein repellents have been synthesized with various chemical formulations and incorporated into composite materials, bonding agents, and denture acrylics to inhibit oral biofilm infections [27,29,30].

In a study by Izumida et al., the effect of coating denture acrylic resin with experimental zwitterion or hydrophilic monomers on the adherence of *C. albicans, Candida glabrata*, and *S. mutans* to the acrylic resin was evaluated [27]. Using metabolic activity and cell viability (CFU) assays, they were able to show significant reduction in absorbance and CFU values compared to control groups [27]. In another study by Lazarin et al., coatings containing hydrophilic monomers 2-hydroxyethyl methacrylate (HEMA), 2-hydroxypropyl methacrylate (HPMA), 2-trimethylammonium ethyl methacrylate chloride (TMAEMC), and zwitterionic monomers at 35% mol fraction significantly decreased *C. albicans* adhesion [17]. In a recent study, coating denture base materials with nano-coat, Optiglaze, or nano-silica materials showed significant reduction in *C. albicans* adhesion and biofilm adhesion. 

MPC is a methacrylate with a phospholipid polar group in its side chain [31,32]. It was previously shown to be highly hydrophilic, with the ability to repel proteins and prevent microbial attachment and colonization [32]. Regarding its mechanism of action, MPC was shown to have high fractions of free water, unlike bound water that could induce protein adsorption [31,32,33]. The large amounts of free water around phosphorylcholine groups can prevent protein adsorption and subsequently reduce microbial attachment [32,33].

In the present study, incorporating MPC at 4.5% into HIPA resin reduced *C. albicans* biofilm CFU by one order of magnitude. Similar results were obtained by Wang et al. when MPC was incorporated into a composite resin and tested for its antibacterial properties against periodontal bacteria [33]. Zhang et al. incorporated MPC into resin-modified glass ionomer cement (RMGI) for orthodontic applications [34]. Using a dental plaque microcosm biofilm model, they showed that RMGI with 3% MPC had reduced protein adsorption, bacterial adhesion, lactic acid production, and CFU counts compared with the control. In addition, this antibacterial effect was maintained after 30 days of water ageing [34]. When MPC was incorporated into an adhesive system, it significantly reduced protein adsorption and bacterial adhesion, resulting in total microorganisms, total streptococci, and *S. mutans* CFU that were an order of magnitude lower than that of the control [35].

An important property of denture materials is their roughness measurements. Rough denture surfaces accumulate more plaque and oral proteins, promoting microbial colonization and proliferation. Adhesion of *C. albicans* is promoted by rough surfaces and has been shown to play an important role in the pathogenesis of DS [36,37]. In the present study, incorporating MPC at its highest weight percentage of 4.5% did not influence the roughness measurements compared with the control group with 0% MPC. Thus, we concluded that 4.5% MPC has minimal to no effect on denture surface roughness. 

In addition, it was previously shown that fluctuations in environmental pH can influence microbial growth and survival [36]. Lactic acid produced by cariogenic bacteria can also cause tooth demineralization, especially in over-denture abutments and root surfaces [37]. For that reason, the effect of adding MPC into HIPA resin on solution pH was investigated. All experimental groups with MPC percentages ranging from 1.5% to 4.5% showed no difference in solution pH values and demonstrated neutral pH values (pH ≅ 7). Therefore, incorporating MPC into acrylic denture materials shows promise for preventing protein adsorption, microbial adhesion, and potentially DS, without jeopardizing other needed properties. Future studies should focus on other properties, like hardness measurements and flexural strength, of the newly developed material. In addition, a more complex biofilm model mimicking clinical conditions should be used to provide a more clinically relevant idea about the material’s potential clinical performance. 

## 5. Conclusions

An antimicrobial denture acrylic resin was developed through the incorporation of MPC for its protein-repelling properties. Denture acrylic resin containing higher concentrations of MPC significantly reduced *C. albicans* CFU by one log without compromising the surface roughness or causing adverse effects on solution pH. This newly developed denture acrylic material has the potential to prevent oral microbial infections, such as denture stomatitis, and improve the quality of life of denture-wearing individuals.

## Data Availability

The data presented in this study are available on request from the corresponding author.
